# A Globally Accurate Neural Network Potential Energy Surface and Quantum Dynamics Studies on Be^+^(^2^S) + H_2_/D_2_ → BeH^+^/BeD^+^ + H/D Reactions

**DOI:** 10.3390/molecules29143436

**Published:** 2024-07-22

**Authors:** Zijiang Yang, Furong Cao, Huiying Cheng, Siwen Liu, Jingchang Sun

**Affiliations:** School of Physics and Electronic Technology, Liaoning Normal University, Dalian 116029, China

**Keywords:** potential energy surface, quantum dynamics, ab initio, neural network, time-dependent wave packet

## Abstract

Chemical reactions between Be^+^ ions and H_2_ molecules have significance in the fields of ultracold chemistry and astrophysics, but the corresponding dynamics studies on the ground-state reaction have not been reported because of the lack of a global potential energy surface (PES). Herein, a globally accurate ground-state BeH_2_^+^ PES is constructed using the neural network model based on 18,657 ab initio points calculated by the multi-reference configuration interaction method with the aug-cc-PVQZ basis set. On the newly constructed PES, the state-to-state quantum dynamics calculations of the Be^+^(^2^S) + H_2_(*v*_0_ = 0; *j*_0_ = 0) and Be^+^(^2^S) + D_2_(*v*_0_ = 0; *j*_0_ = 0) reactions are performed using the time-dependent wave packet method. The calculated results suggest that the two reactions are dominated by the complex-forming mechanism and the direct abstraction process at relatively low and high collision energies, respectively, and the isotope substitution has little effect on the reaction dynamics characteristics. The new PES can be used to further study the reaction dynamics of the BeH_2_^+^ system, such as the effects of rovibrational excitations and alignment of reactant molecules, and the present dynamics data could provide an important reference for further experimental studies at a finer level.

## 1. Introduction

Chemical reactions of alkaline earth metal ions with neutral molecules have been extensively studied in the past several decades because of their crucial role in quantum information [1,2], cold molecules [3,4,5,6], single ion reactions [7,8,9,10], astrophysics [11,12], and other frontier fields. Among those ion-molecule systems, the reactive collisions between Be^+^ ions and H_2_ molecules or their isotopic variations, as the simplest example, have obtained increasing attention both experimentally and theoretically. One main reason is that singly ionized beryllium presents unique advantages in sympathetically cooling other atomic or molecular ions in Penning traps [13,14]; previously, it was usually used for carrying quantum bits in quantum information processing [15,16]. Moreover, the BeH^+^ molecule, mainly formed by the Be^+^ + H_2_ reaction, has been identified in stars and comets, so understanding its formation is also significant for the evolution of interstellar molecules [17,18].

Several experimental studies on the reactions between laser-cooled Be^+^ ions and molecular hydrogen or its isotopes have been carried out. Roth et. al. studied the reactions of ultracold Be^+^(^2^P) ions with room-temperature H_2_/D_2_/HD molecules by using the laser-cooling ion trap apparatus [9]. They used secular excitation mass spectroscopy to verify the product molecules, and the reaction rate constants were determined from the time evolution of the number of Be^+^ ions or the spontaneous emission fluorescence rate. The measured rate coefficients of the reactions of laser-cooled Be^+^(^2^P) with H_2_, D_2_, and HD molecules can reach 1.3 × 10^−9^, 1.3 × 10^−9^, and 1.1 × 10^−9^cm^3^/s, respectively, which is in agreement with the results of molecular dynamics simulations based on Langevin ion-neutral reaction model. In addition, the ultracold product BeH^+^ and BeD^+^ molecules were prepared by the sympathetic cooling of Be^+^ ions. In 2015, Sawyer et al. demonstrated photodissociation results exploiting the B^1^Π ← X^1^Σ^+^ electronic transition in BeH^+^ molecules within a Coulomb crystal of thousands of Be^+^ ions bound in a Penning trap [15]. The BeH^+^ molecules are obtained by the reactions of trapped Be^+^(^2^P) ions with background H_2_ molecules within the vacuum chamber, which act as a primary impurity species in large-scale trapped-ion quantum information experiments. A rotational-state-insensitive dissociation scheme was proposed to produce Be^+^ ions and H atoms, thereby achieving the restoration of Be^+^ ions without reloading. These experimental studies on the Be^+^(^2^P) + H_2_ reaction have opened new possibilities for high-precision measurements, chemical reactions at ultracold temperature, and coherent manipulation of quantum states.

In the theoretical aspect, the ab initio calculations on the BeH_2_^+^ molecular system have a long history, and the quantum dynamics studies on the electronically excited Be^+^(^2^P) reacting with H_2_ and its isotopic variations were also reported in recent years. In 1971, Poshusta et al. computed the first ab initio potential energy surface (PES) in *C*_2*v*_ symmetry using the self-consistent-field theory and valence bond configuration interaction method [19]. Both calculations predicted that this system is weakly bound in the 1^2^*A*_1_ state with a binding energy of 0.19 eV relative to the Be^+^(^2^S) + H_2_ asymptotic region, and the binding energy in the 1^2^*B*_2_ state is around 3.2 eV. Raimondi and Gerratt used the spin-coupled VB method to study the ground-state and several low-lying excited states PESs correlated with the reactions of Be^+^ + H_2_ → BeH^+^ + H, BeH + H^+^ [20]. According to the calculation results, they found that the reactive process of Be^+^(^2^S) + H_2_(*X*^1^Σ*_g_*^+^) → BeH^+^(*X*^1^Σ^+^) + H(^2^S) is endoergic by around 1.57 eV. Artiukhin et al. mapped a three-dimensional ground-state BeH_2_^+^ PES based on the reproducing kernel Hilbert space method to fit the ab initio points calculated at the CCSD(T) level, and the structural and spectroscopic characteristics of the Be^+^−H_2_ and Be^+^−D_2_ complexes are discussed in detail [21]. In 2018, we constructed the first global BeH_2_^+^ diabatic potential energy matric (DPEM) that involved the lowest two electronic states (1^2^A′ and 2^2^A′) by combining a molecular property-based diabatization scheme and the artificial neural network (NN) model [22]. On this DPEM, the quantum dynamics calculations of the non-adiabatic Be^+^(^2^P) + H_2_ → BeH^+^ + H reaction were performed, and the results showed that the product BeH^+^ molecule prefers to populate at high vibrational states and tends to backward scattering. In a later quantum dynamics study on the non-adiabatic Be^+^(^2^P) + HD → BeH^+^/BeD^+^ + D/H reaction [23], the results present distinct dynamics behaviors on the two product channels, which can be attributed to the avoided crossing effects of the diagonal term of the DPEM. This research has explained the microcosmic dynamics mechanisms of the strong intramolecular isotope effects presented in the previous experimental studies on the similar reactive systems of the Mg^+^(^2^P) + HD [24] and Ca^+^(^2^P) + HD [7]. Recently, Guan et al. structured a new DPEM for the two lowest states of the Be^+^(^2^P) + H_2_ reactive system based on the combined-hyperbolic inverse-power-representation method in which the permutation inversion symmetry of complete nuclear is correctly retained by adding symmetry constraints on the coefficients of polynomials [25].

Previous experimental studies and dynamics calculations on the BeH_2_^+^ mainly focused on the excited state reaction due to the exothermicity, whereas the dynamics data and microscopic process of the ground-state Be^+^(^2^S) + H_2_ reaction have not been reported up to now, so the understanding of this reaction system is not complete. Implementing rigorous quantum dynamics calculations on a high-fidelity PES can not only give detailed reaction processes and accurate dynamics data but also provide important references for the corresponding experiments at a finer level. However, the previously reported ground-state BeH_2_^+^ PESs cannot be used for reaction dynamics studies because some regions where the reaction can reach are not included. To this end, herein, a globally accurate adiabatic ground-state BeH_2_^+^ PES is constructed using the NN method based on a mass of high-level ab initio points. Furthermore, the quantum dynamics calculations of the Be^+^(^2^S) + H_2_(*v*_0_ = 0, *j*_0_ = 0) → BeH^+^ + H and Be^+^(^2^S) + D_2_(*v*_0_ = 0, *j*_0_ = 0) → BeD^+^ + D reactions at the state-to-state level are carried out using the time-dependent wave packet (TDWP) method to study the dynamics processes and isotope substitution effects in detail. 

## 2. Results and Discussion

### 2.1. Topographic Characteristics of PES

**Figure 1:**
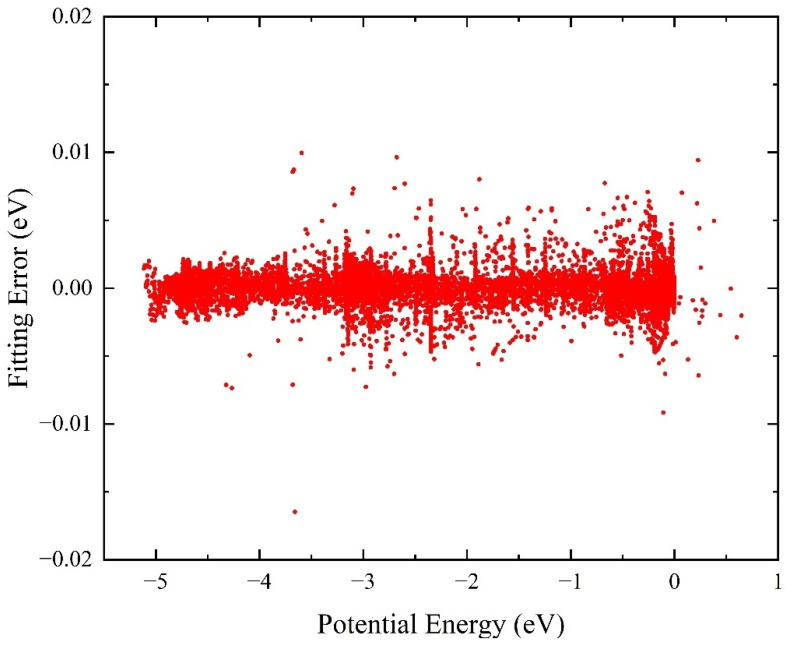
Distribution of the fitting errors of 18,657 ab initio energy points for the ground-state BeH_2_^+^ PES.

The accuracy of NN PES can be measured by the fitting error distribution and the root-mean-square error (RMSE) of all the training points. To ensure the globality of the constructed PES, a mass of 18,657 ab initio points that cover the whole configuration space are selected to participate in the fitting. Figure 1 displays the distribution of the NN fitting errors defined by the difference between the original ab initio results and the fitting values obtained on the analytical ground-state BeH_2_^+^ PES, and the zero of energy value corresponds to the triatomic Be^+^-H-H dissociation limit. It can be seen that the fitting NN PES keeps an extremely small predictive error in the whole energy space, especially for the low potential energy region. The overall RMSE of all the selected 18,657 ab initio points is only 1.03 meV, and the maximum absolute error is 16.5 meV. The percentage of points with an absolute error of less than 5 meV can reach 99.6%, and the corresponding percentage of less than 1 meV is 83.1%. Therefore, the fitting NN PES is accurate enough for the dynamics studies on the Be^+^(^2^S) + H_2_ reactive system. In addition, the NN PES also has strong generalization performance because the data set includes 10% test points. 

**Figure 2:**
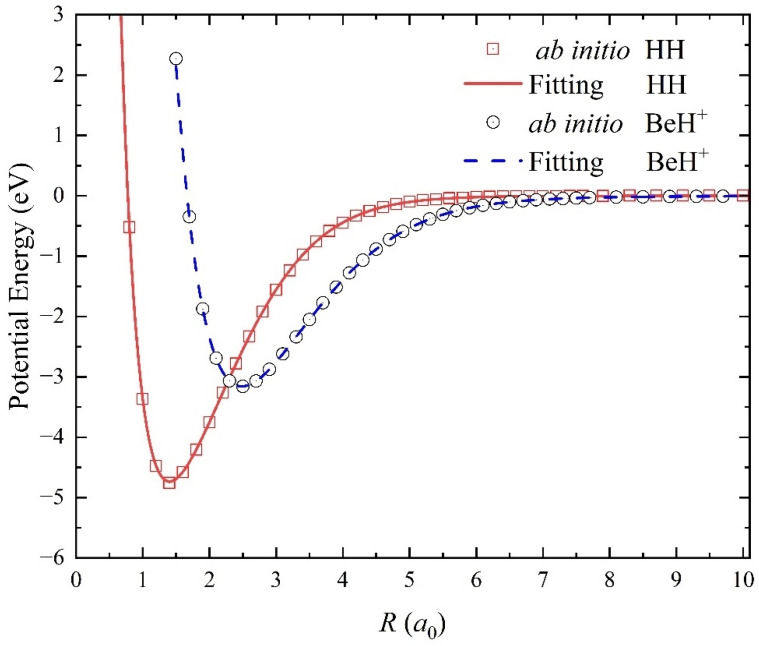
Comparison of the PECs of H_2_(X^1^Σ_g_^+^) and BeH^+^(X^1^Σ^+^) between the results obtained on the ground-state BeH_2_^+^ PES and the original ab initio data.

Figure 2 shows the diatomic potential energy curves (PECs) of H_2_(X^1^Σ_g_^+^) and BeH^+^(X^1^Σ_g_) molecules obtained on the NN PES, compared with the ab initio data. It is clear that the two-body PECs of reactant and product molecules can reproduce the original ab initio results well. To make a further comparison, the molecular constants of equilibrium distance *R_e_*, dissociation energy *D*_e_, vibrational frequencies *ω_e_*, and anharmonicity coefficients *ω_e_x_e_* of the two diatomic molecules are presented in Table 1. The approach for calculating the molecular constants is to keep the third atom away from the diatomic molecule to obtain data points on the analytical PES and then to fit the two-body PECs based on these points using the least squares method. The molecular data of H_2_(X^1^Σ_g_^+^) and BeH^+^(X^1^Σ_g_) molecules calculated on the NN PES are in good agreement with the previous experimental results [26], meaning that the fitting PES are sufficiently reliable for describing the structures and rovibrational states of the reactant and product molecules. 

**Figure 3:**
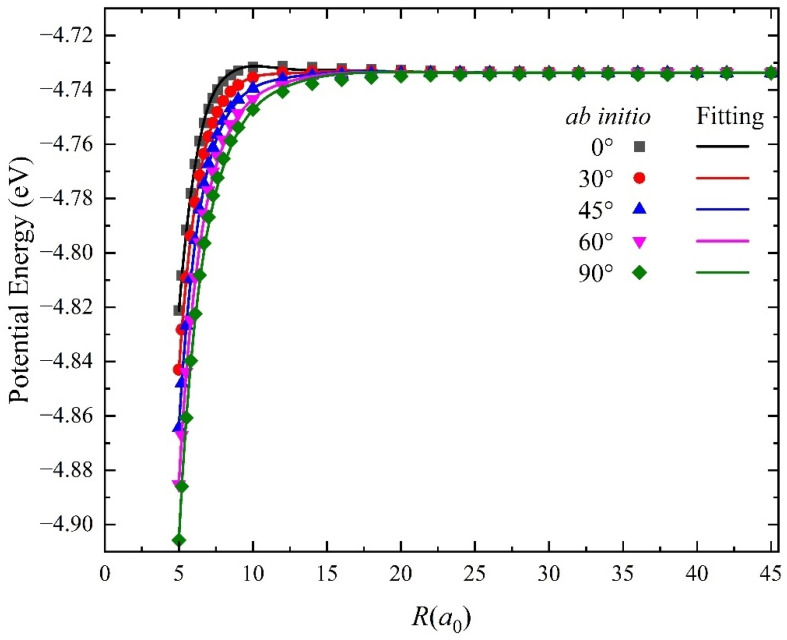
Comparison of the long rang potential obtained on the ground-state BeH_2_^+^ PES and the corresponding ab initio data in the reactant channel as a function of *R* at five Jacobi angles (*θ* = 0°, 30°, 45°, 60° and 90°), and the bond length of HH is fixed at 1.402 *a_0_*.

In the training of NN, the risk of overfitting will markedly increase when the training error decreases to a small value, especially for the long-range potential due to the simpler function relationship between the potential energies and molecular coordinates. As such, we applied the cross-validation method to examine whether the overfitting occurs during the training process. For the ion-molecule reactions, the long-range potential of the reactant channel also plays a crucial role in the calculated dynamics results, and its minor error could generate distinct dynamics features. Figure 3 shows a comparison between the long-range potential obtained on the NN PES and the corresponding ab initio values along the radial direction in the reactant Jacobi coordinates at five selected insertion angles *θ* = 0°, 30°, 45°, 60° and 90°, in which the H_2_ molecule is fixed at its equilibrium bond distance (1.402 *a*_0_). To ensure the fitting NN PES is globally reliable and examine the long-range interaction of the Be^+^(^2^S) + H_2_ reactive system, the radial distance *R* is calculated up to 45 *a*_0_. It is clear that the long-range potentials of the fitting PES are extremely smooth and accord quite well with the original high-precision ab initio data for each insertion angle, and there is no interaction between the Be^+^ ion and H_2_ molecule when *R* is greater than 30 *a*_0_. The fitting value of all long-range ab initio points is less than 1 meV, which almost does not influence the dynamics results for this endothermic reaction system. Therefore, there are no overfitting behaviors during the NN fitting and the constructed PES are sufficiently accurate for describing the long-range interactions.

**Figure 4:**
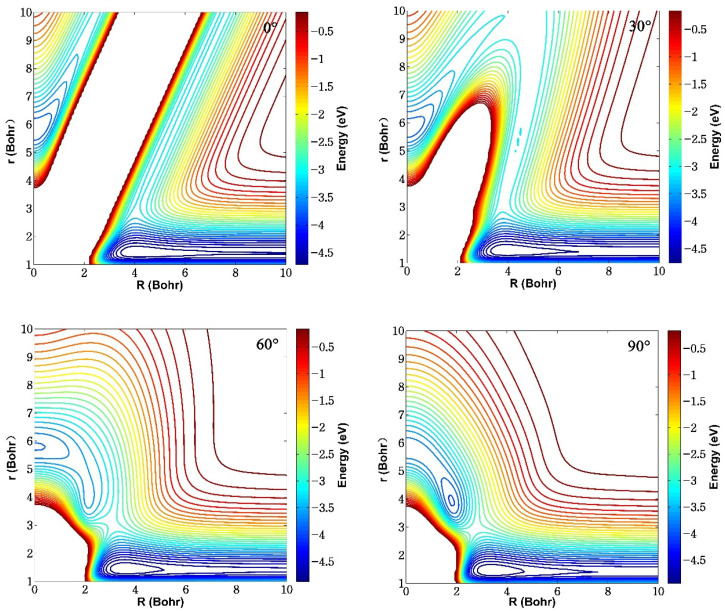
Contours of the ground-state BeH_2_^+^ PES fixed at four different insertion angles (*θ* = 0°, 30°, 60°, and 90°) in the reactant Jacobi coordinates.

Figure 4 gives the contour plots of the ground-state BeH_2_^+^ PES fixed at four different insertion angles (*θ* = 0°, 30°, 60°, and 90°) in the reactant Jacobi coordinates, *r* and *R* represent the bond length of HH and the distance between the Be^+^ ion and the center of mass of the H_2_ molecule, respectively, and *θ* is the angle between *r* and *R*. The energy values are relative to the triatomic Be^+^-H-H dissociation limit, and the maximum potential energy is set as 0 eV to clearly show the structures of wells and barriers. There exists a valley on the bottom corresponding to the reactant channel of Be^+^(^2^S) + H_2_ for each insertion angle, which is located at *r* = 1.402 *a*_0_, and the energy is equal to the dissociation energy of the H_2_ molecule. An obvious potential well can be found and the depth of the well gradually becomes deeper as the increase of insertion angle. When *θ* = 90°, the BeH_2_^+^ system is at *C_2v_* symmetry, and the well with the energy value of −0.374 eV relative to the Be^+^(^2^S) + H_2_ asymptotic region is located at *r* = 1.45 *a*_0_, *R* = 3.38 *a*_0_, which is also the global minimum energy configuration of the ground-state BeH_2_^+^. Therefore, the reaction proceeds along a T-shaped pathway at relatively low collision energy. The equilibrium structure and potential energy are consistent with the previous high-level ab initio results (*r* = 1.45 *a*_0_, *R* = 3.35 *a*_0_, *E* = −0.374 eV) [21], suggesting that the fitting PES can accurately describe the interaction region. In addition, there is a barrier between the reactant channel and the well, and the energy value of the barrier decreases as the angle increases, which could be formed by the avoided crossing effects of the first excited state 2^2^A′.

**Figure 5:**
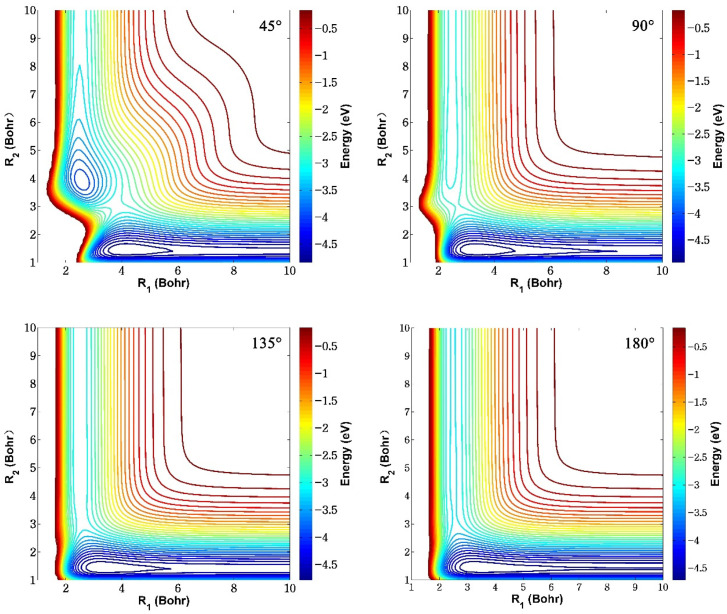
Contours of the ground-state BeH_2_^+^ PES fixed at four different Be^+^-H-H angles (45°, 90°, 135°, and 180°).

Figure 5 displays the contour maps of the ground-state BeH_2_^+^ PES at four different fixed Be^+^-H-H angles (45°, 90°, 135°, and 180°). *R*_1_ and *R*_2_ represent the bond lengths of Be^+^-H and H-H, respectively. It can be seen that the fitting PES is very smooth in the entire configuration space and no non-physical well or barrier is presented for each approach angle, implying that the constructed PES is globally accurate and no overfitting occurs. There exist two valleys on the bottom and left, corresponding to the reactant Be^+^(^2^S) + H_2_ channel and product BeH^+^ + H channel, respectively. The energy value of the left channel is larger than the one of the bottom channel, meaning that the Be^+^(^2^S) + H_2_ is endothermic. The reactant channel and product channel are connected by a shallow well and an obvious barrier, and the depth of the well and height of the barrier gradually decreases as the Be^+^-H-H angle increases. The above two sets of contour plots present multiple potential wells and saddle point structures, suggesting that the topography characteristics of ground-state BeH_2_^+^ PES are relatively complex.

**Figure 6:**
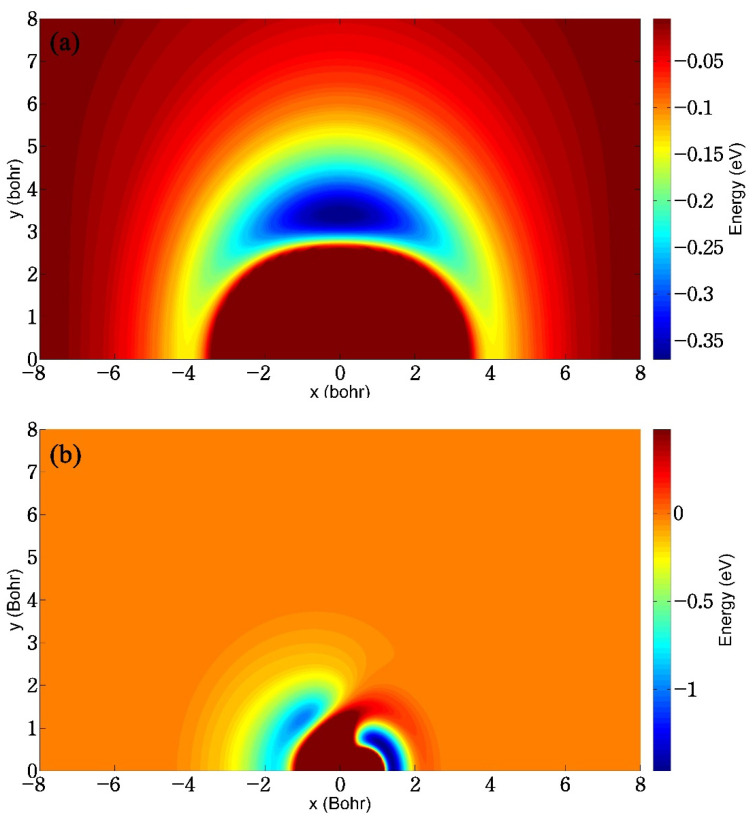
(**a**) Contour plot of the ground-state BeH_2_^+^ PES for the Be^+^ ion moving around the H_2_ molecule with the bond length fixed at 1.402 *a*_0_; (**b**) Contour plot of the ground-state BeH_2_^+^ PES for the H atom moving around the BeH^+^ molecule with the bond length fixed at 2.487 *a*_0_.

Figure 6a shows the contour plot of the fitted ground-state BeH_2_^+^ PES in the case of the Be^+^ ion moving around the HH molecule that is fixed at its equilibrium bond length of 1.401 *a*_0_, and the energy is equal to zero when the Be^+^ ion is far from the H_2_ molecule. This map shows that the constructed PES has excellent exchange symmetry about the two identical H atoms. It can be seen that the Be^+^ ion is always attracted by the H_2_ molecule, and there is a well with a depth of 0.37 eV at the mid perpendicular of HH, indicating that the title reaction prefers to proceed along an insertion pathway at a relatively low collision energy. A similar contour to Figure 6a but for an H atom moving around the BeH^+^ molecule is displayed in Figure 6b. The BeH^+^ bond length is fixed at its equilibrium distance of 2.487 *a*_0_ and the energy is set as zero when the H atom is far from the BeH^+^ molecule. Different from the case of Figure 6a, the H atom is mainly subject to repulsive interactions of the BeH^+^ molecule, and attractive potentials appear when they get close to each other. It is clear that the well around the H atom is shallower than the one around the Be^+^ ion, implying that this H atom is more easily bounced away from the side of H in the product region.

**Figure 7:**
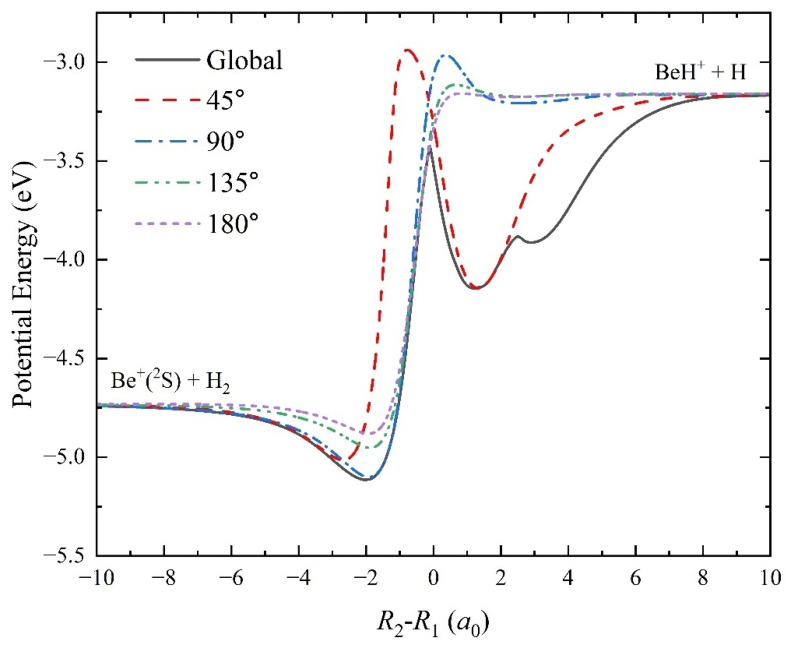
Global MEP and the MEPs of the Be^+^(^2^S) + H_2_ → BeH^+^ + H reaction at four Be^+^-H-H approach angles (45°, 90°, 135°, and 180°) calculated on the ground-state BeH_2_^+^ PES.

To show the characteristics of the Be^+^(^2^S) + H_2_ reaction system by the topography of PES more clearly, the global minimum energy path (MEP) and the MEPs of the title reaction at four different Be^+^-H-H approach angles (45°, 90°, 135°, and 180°) are presented in Figure 7. The MEPs are calculated by scanning the fitting ground-state BeH_2_^+^ with the step lengths of Δ*R* = 0.01 *a*_0_ and Δ∠Be^+^-H-H = 1° at different reactant coordinates of *R*_2_ − *R*_1_ to search the minimum energy value. The global MEP features a shallow well with a depth of 0.374 eV and a barrier with an energy value of 1.317 eV relative to the Be^+^(^2^S) + H_2_ asymptotic region. The molecular system is at *C*_2*v*_ symmetry for the well, which also corresponds to the global minimum energy structure shown in Figure 4. The molecular structure of the barrier is at the collinear *C*_∞*v*_ symmetry (*R*_1_ = 2.50 *a*_0_, *R*_2_ = 2.39 *a*_0_), which is also the transition state of the Be^+^(^2^S) + H_2_ → BeH^+^ + H reaction channel. In addition, there is a sharp structure on the global MEP, which is not present on the other MEPs of the fixed approach angle. Near the geometries of the transition state, the non-adiabatic couplings between the ground-state 1^2^A′ and the first excited state 2^2^A′ are strong enough, which has been shown in previous diabatic PES [22], thus this sharp is formed by the avoided crossing effect of the 2^2^A′ electronic state. There also exists a small sharp at *x* = 2.51 *a*_0_, which corresponds to the saddle point shown in Figure 4. At relatively low collision energy, the Be^+^ ion approaches the H_2_ molecule along a T-shaped pathway with the elongation of the HH bond to form the triatomic BeH_2_^+^complex; then, a collinear intermediate Be^+^-H-H is generated before the formation of the product molecule; finally, the HH bond is broken to produce the BeH^+^ molecule and H atom. For the MEP of fixed Be^+^-H-H approach angle, there is also a well and a barrier that is higher than the product channel. As the angle increases, the depth of the well and the height of the barrier decrease, implying that the lifetime of the complex becomes shorter and the reaction threshold becomes lower. Therefore, the reaction more easily occurs at a large Be^+^-H-H collision angle. When the vibrational zero-point energies of H_2_ and BeH^+^ molecules are included, the endothermicity of the Be^+^(^2^S) + H_2_ (*v*_0_ = 0, *j*_0_ = 0) → BeH^+^ + H reaction determined by the PES is 1.715 eV. 

### 2.2. Quantum Dynamics

**Figure 8:**
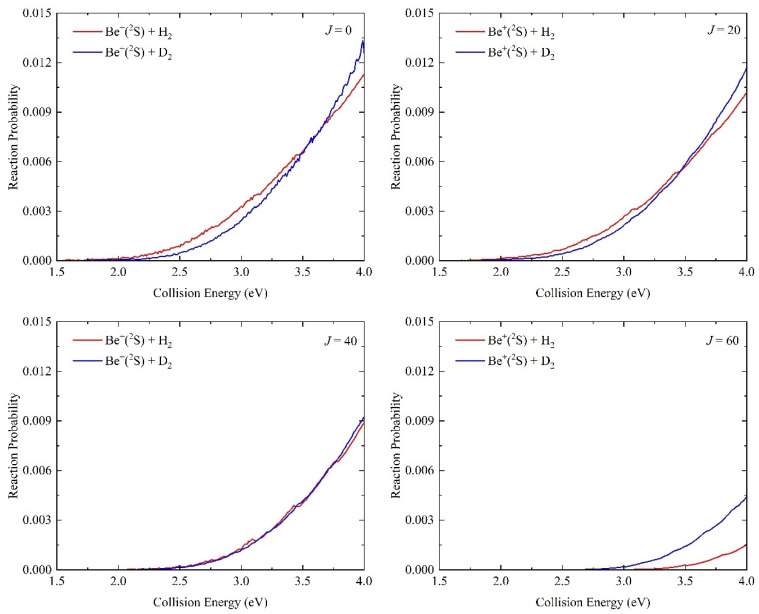
Total reaction probabilities as a function of collision energy for the Be^+^(^2^S) + H_2_(*v*_0_ = 0, *j*_0_ = 0) and Be^+^(^2^S) + D_2_(*v*_0_ = 0, *j*_0_ = 0) reactions with four partial waves (*J* = 0, 20, 40, and 60) calculated by the TDWP method on the ground-state BeH_2_^+^ PES.

Based on the newly constructed NN PES, the quantum dynamics calculations of the Be^+^(^2^S) + H_2_(*v*_0_ = 0, *j*_0_ = 0) and Be^+^(^2^S) + D_2_(*v*_0_ = 0, *j*_0_ = 0) reactions are performed at the state-to-state level using the TDWP method to study the microscopic dynamics processes. The collision energy dependence of total reaction probabilities of the Be^+^(^2^S) + H_2_(*v*_0_ = 0, *j*_0_ = 0) and Be^+^(^2^S) + D_2_(*v*_0_ = 0, *j*_0_ = 0) reactions with four different partial waves (*J* = 0, 20, 40, and 60) are shown in Figure 8. For *J* = 0, the threshold of the Be^+^(^2^S) + H_2_ process is consistent with the endothermicity calculated on the NN PES because the global MEP is barrierless, and it is slightly smaller than the one of the Be^+^(^2^S) + D_2_ reaction; this is because the smaller difference of zero-point energies between the reactant and product for the later. There exist some oscillation peaks on the reaction probability curves since the formation of a short-lived complex at the shallow wells on the MPEs, which can support the bound and quasi-bound states, and the resonance structures are gradually smoothed as the *J* value increases, which is attributed to the increasing centrifugal barrier decreasing the effect of well; as a result, the product can be generated without the BeH_2_^+^intermediate. The centrifugal potential can also cause the probability value to become smaller, and the Be^+^(^2^S) + D_2_ reaction features a slower falling rate than the Be^+^(^2^S) + H_2_ because the former has a larger reduced mass. 

**Figure 9:**
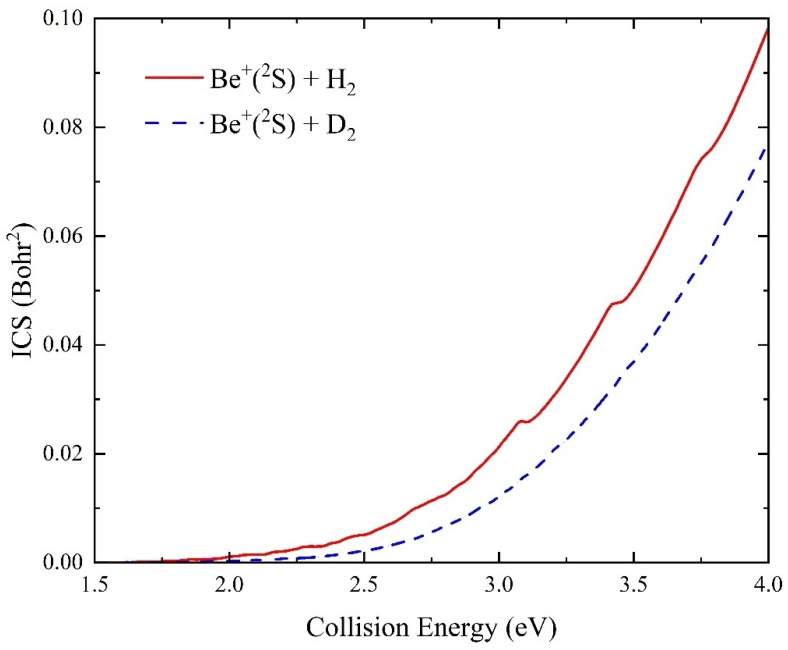
Total ICSs as a function of collision energy for the Be^+^(^2^S) + H_2_(*v*_0_ = 0, *j*_0_ = 0) and Be^+^(^2^S) + D_2_(*v*_0_ = 0, *j*_0_ = 0) reactions calculated by the TDWP method on the ground-state BeH_2_^+^ PES.

Figure 9 displays the total integral cross sections (ICSs) of the Be^+^(^2^S) + H_2_(*v*_0_ = 0, *j*_0_ = 0) and Be^+^(^2^S) + D_2_(*v*_0_ = 0, *j*_0_ = 0) reactions as a function of collision energy. Compared to the reaction probabilities, the ICS curves are very smooth because of the summing effects of all the available partial waves. In the studied collision energy range, the ICSs of the two reactions monotonously rise with the increase of collision energy, which conforms to the characteristic of an endothermic reaction. The ICS value of the Be^+^(^2^S) + H_2_ reaction is larger than the Be^+^(^2^S) + D_2_ reaction due to the smaller initiating energy. However, their difference becomes large with the increase of collision energy, indicating that the dominance of high partial waves on the Be^+^(^2^S) + D_2_ reaction is more obvious, causing the ICS value to rise more slowly. 

**Figure 10:**
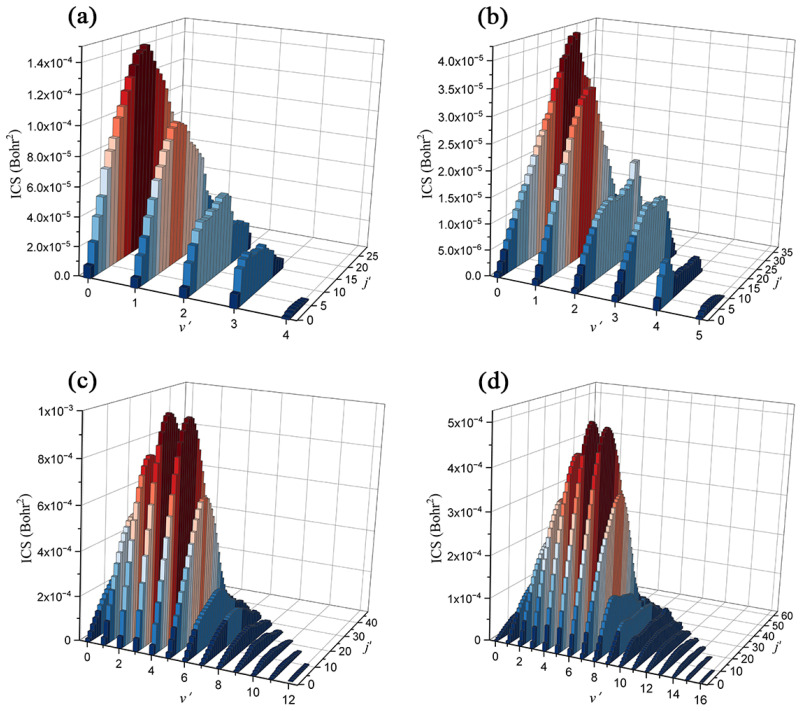
Rovibrationally state-resolved ICSs of the Be^+^(^2^S) + H_2_(*v*_0_ = 0, *j*_0_ = 0) reaction at (**a**) 2.5 eV and (**b**) 4.0 eV collision energies and the Be^+^(^2^S) + D_2_(*v*_0_ = 0, *j*_0_ = 0) reaction at (**c**) 2.5 eV and (**d**) 4.0 eV collision energies calculated by the TDWP method on the ground-state BeH_2_^+^ PES.

To further study the Be^+^(^2^S) + H_2_(*v*_0_ = 0, *j*_0_ = 0) and Be^+^(^2^S) + D_2_(*v*_0_ = 0, *j*_0_ = 0) reactions at the state-to-state level, the rovibrationally state-resolved ICSs of the product molecules of the two reactions at the collision energies of 2.5 eV and 4.0 eV are presented in Figure 10. Compared to the product BeH^+^ molecule, the BeD^+^ product can populate at higher vibrational and rotational states for each collision energy even though a larger endothermicity of the Be^+^(^2^S) + D_2_ channel. This is because the BeD^+^ molecule features a smaller vibrational frequency and rotational constant than the BeH^+^ molecule, resulting in the adjacent rovibrational energy level difference of the former being smaller. At *E*_C_ = 2.5 eV, the maximum available vibrational and rotational quantum numbers of the BeH^+^ and BeD^+^ products are (4, 29) and (5, 38), respectively. The high vibrational state corresponds to the smaller ICS value for both product molecules, whereas the peaks of distribution of rotational state are located at relatively high rotational states. This vibrationally cold and rotationally hot quantum state distribution suggests the two reactions are dominated by the complex-forming mechanism. The reactions are dominated by the low partial waves and mainly proceed along the global MEP when the collision energy is slightly higher than the corresponding reactive thresholds, thus a short-lived complex can be formed in the shallow well before the formation of the product molecule. It can be seen that the BeD^+^ molecules prefer to populate at a higher rotational state since the zero point energy of the intermediate BeD_2_^+^ is smaller than the one of BeH_2_^+^, so the effective well of the Be^+^(^2^S) + D_2_ is deeper, causing the forming complex to have a longer lifetime. As the collision energy increases to 4.0 eV, the more available rovibrational states are opened, and the highest vibrational and rotational quantum numbers for the BeH^+^ and BeD^+^ are (4, 29) and (12, 48), respectively. Different from the case of 2.5 eV collision energy, there exists obvious vibrational population inversion for the product molecules of the two reactions, namely the products prefer to distribute at relatively high vibrational states, while the ICS values are very small for the low vibrational state of *v’* = 0 or 1. This is because more reaction paths with shallower wells, shown in Figure 7, are gradually opened up as the increase of collision energy. The effects of high partial waves also smooth the effective well of the global MEP, leading to the reactions being dominated by a direct abstraction approach. Compared to the BeH^+^ molecule, the population inversion of the BeD^+^ is more obvious, which can be attributed to the more prominent effects of high partial waves 

**Figure 11:**
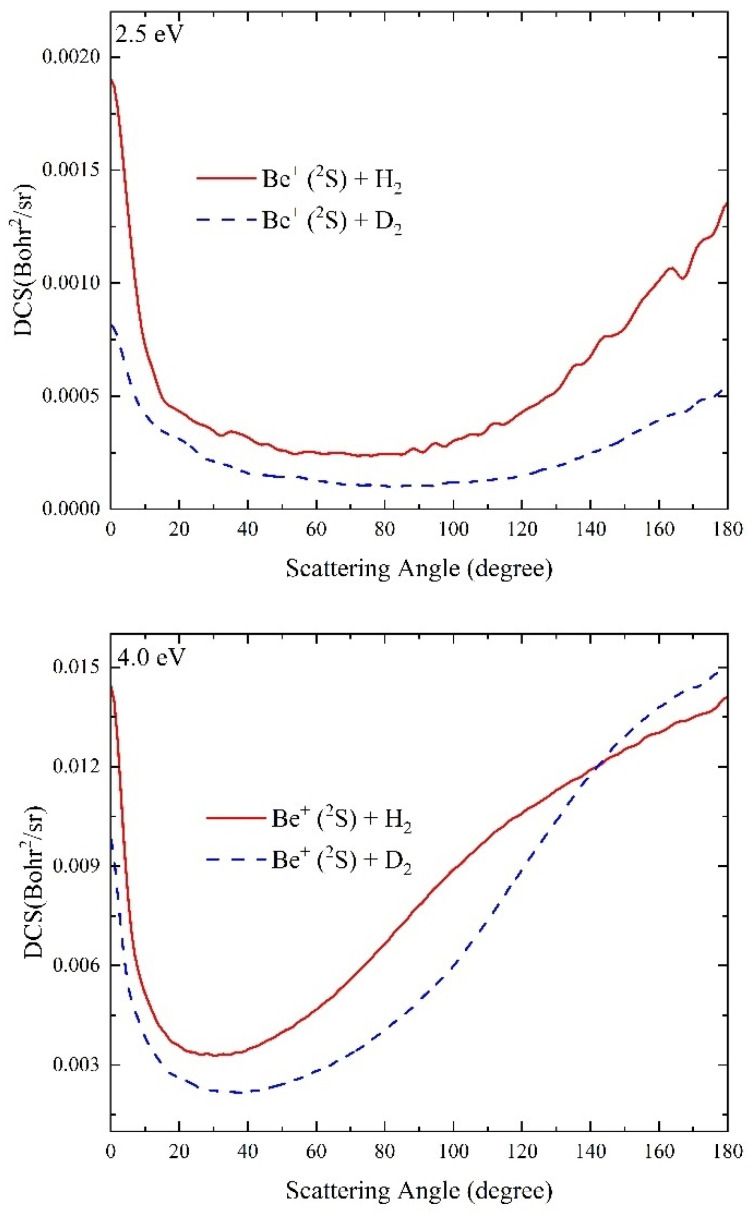
Total DCSs of the Be^+^(^2^S) + H_2_(*v*_0_ = 0, *j*_0_ = 0) and Be^+^(^2^S) + D_2_(*v*_0_ = 0, *j*_0_ = 0) reactions at 2.5 eV and 4.0 eV collision energies calculated by the TDWP method on the ground-state BeH_2_^+^ PES.

The differential cross sections (DCSs) can present the dynamics mechanisms more intuitively by giving the scattering angular distributions of product molecules. Figure 11 displays the total DCSs of the Be^+^(^2^S) + H_2_(*v*_0_ = 0, *j*_0_ = 0) and Be^+^(^2^S) + D_2_(*v*_0_ = 0, *j*_0_ = 0) reactions at the collision energies of 2.5 and 4.0 eV. At *E*_C_ = 2.5 eV, the peaks of the DCS curves are located at the two extreme angles (0° and 180°) for both two reactions, and the angular distributions are nearly symmetric concerning 90°, showing the typical feature of a complex-forming reaction. As discussed above, the global MEP dominates the collision processes when the collision energy is slightly larger than the corresponding reaction threshold values. Compared to the Be^+^(^2^S) + H_2_ reaction, the Be^+^(^2^S) + D_2_ reaction features a better forward-backward scattering symmetry, which is because the effective well of the latter is shallower, meaning that the forming complex has a longer lifetime. When the collision energy increases to 4.0 eV, the forward-backward symmetry angle distributions are broken for both two reactions, showing the obvious non-statistical behaviors, and the backward scattering plays the dominant role in the two collision processes. This is also consistent with the discussion above. As the collision energy increases, the well on the global MEP is smoothed, and the other collision paths without a well are gradually opened up, so the product molecules are generated by a direct abstraction approach. In general, the isotope substitution has little effect on the dynamics behaviors of the Be^+^(^2^S) + H_2_(*v*_0_ = 0, *j*_0_ = 0) reaction, except for the difference in the dynamics data. 

## 3. Theoretical Methods 

### 3.1. Ab Initio Calculations

The ab initio points of the ground-state (1^2^A′) BeH_2_^+^ are calculated by the internally contracted multi-reference configuration interaction (icMRCI) method [27,28] with the Davidson correction (+Q), and the aug-cc-PVQZ basis set [29] is used for H atom and Be atom, respectively. The molecular orbitals are optimized by the complete active space self-consistent field (CASSCF) method as the reference wavefunctions. In the CASSCF calculations, three electronic states of 1^2^A′, 2^2^A′, and 1^2^A″ are equal-weight, and the three valence electrons are included in 16 active orbitals (12a′ + 4a″), corresponding to the 1*s*, 2*s,* and 2*p* orbitals of H and 1*s*, 2*s*, 2*p*, and 3*s* orbitals of Be atom. A mass of molecular configurations over a great range of space within *C_s_* symmetry are selected to ensure that the fitting PES can entirely cover the dynamics-relevant regions. The geometries are selected in the reactant Jacobi coordinates, constructed by 0.8 ≤ *r*/*a*_0_ ≤ 30.0, 0.1 ≤ *R*/*a*_0_ ≤ 45.0, 0 ≤ *θ* ≤ π/2. All the ab initio calculations are implemented by utilizing Molpro 2012 program [30].

### 3.2. NN Fitting

There is increasing attention to representing molecular PESs by machine learning algorithms, such as NN [31,32,33,34,35,36,37,38,39] and kernel-based Gaussian process regression [40,41,42,43,44,45,46,47] models. Among those methods, the artificial NN model features strong generalization performance and high fitting accuracy and has become one of the most popular schemes in representing globally reactive PESs for simple systems. In this work, the backpropagation NN method is used to structure the global ground-state BeH_2_^+^ PES. To ensure the fitting PES satisfies the exchange symmetry about the two same H atoms, the permutation invariant polynomials (PIPs) [48,49] are constructed by the bond length between two atoms. The primary invariants can be written as:

(1) $$p_{i}=\exp(-\alpha R_{i}),(i=1,2,3)$$

where *α* is an adjustable parameter. Next, the symmetrized polynomial vector *S* = {*S_i_*} is constructed as:

(2) $$S_{1}=(p_{1}+p_{3})/2$$

(3) $$S_{2}=p_{1}\times p_{3}$$

(4) $$S_{3}=p_{2}$$

Finally, the vector *S* is normalized treatment as:

(5) $$I_{i}=\frac{2(S_{i}-S_{i,\min})}{\left(S_{i,\max}-S_{i,\min}\right)}-1,(i=1,2,3)$$

where *S_i_*_,min_ and *S_i_*_,max_ are the minimum and maximum of *S_i_*, respectively. The normalized vector *I* serves as the input of NN, and the output is the corresponding normalized potential energy. The input and output are linked by two hidden layers with 15 neurons in each layer. The hyperbolic tangent function is selected as the transfer function *φ* for the 1–2 and 2–3 layers, and the 3–4 layer is connected by a simple linear function, so the analytical expansion of the fitting global PES is written as:

(6) $$V_{\mathrm{fit}}=\varphi^{\left(3\right)}\left({b_{1}}^{\left(3\right)}+\sum_{i=1}^{15}w_{i1}^{\left(3\right)}\varphi^{\left(2\right)}\left({b_{i}}^{\left(2\right)}+\sum_{j=1}^{15}w_{ji}^{\left(2\right)}\varphi^{\left(1\right)}\left({b_{j}}^{\left(1\right)}+\sum_{k=1}^{3}w_{kj}^{\left(1\right)}I_{k}\right)\right)\right)$$

Here, the mean squared error between the predictive value and ab initio acts as the cost function to evaluate the performance of the training model, and the linking weight value *w* and bias value *b* between the adjacent two layers are circularly optimized by the Levenberg–Marquardt algorithm [50].

A total of 18,657 ab initio points that cover the entire configuration space are selected to participate in the fitting of the ground-state BeH_2_^+^ PES. To avoid the overfitting behavior, the data points are randomly divided into a 90% training set and a 10% testing set. The training should be stopped immediately when the fitting error of the training set declines slowly or the predictive error of the test set starts to rise, which is usually the overfitting signal. 

### 3.3. TDWP Method

The most reliable approach to obtaining reaction dynamics data theoretically is to carry out rigorous quantum mechanical calculations on a globally accurate PES [51,52]. The TDWP method features high fidelity and strong extensibility and has been widely applied to the quantum dynamics studies of various triatomic systems [53,54,55,56,57,58]. Here, we only give an abbreviated description of the TDWP method, and more details can be referenced in the relevant works [59,60,61].

The Hamiltonian of the Be^+^(^2^S) + H_2_/D_2_ reaction system in the reactant Jacobi coordinates (*r*, *R*, *θ*) can be expressed as

(7) $$\hat{H}=-\frac{\hbar^{2}}{2\mu_{R}}\frac{\partial^{2}}{\partial R^{2}}-\frac{\hbar^{2}}{2\mu_{r}}\frac{\partial^{2}}{\partial r^{2}}+\frac{{\left(\hat{J}-\hat{j}\right)}^{2}}{2\mu_{R}R^{2}}+\frac{{\hat{j}}^{2}}{2\mu_{r}r^{2}}+\hat{V}(r,R,\theta )$$

where *j* and *J* are the quantum numbers of rotational angular momentum of the reactant molecule and the total angle momentum of this system, respectively. *µ_r_* and *µ_R_* are the reduced masses associated with *R* and *r* coordinates, respectively. *V* represents the atom-diatom interaction potential excluding the diatom reference potential. The total wavefunction in the body-fixed representation can be expanded to the translational-vibrational-rotational form, written as

(8) $$\Psi^{JM\varepsilon }(R,r,\theta )=\sum_{nvjK}F_{nvjK}^{JM\varepsilon }D_{MK}^{J\varepsilon }\left(\Omega \right)u_{n}\left(R\right)\phi_{v}(r)y_{jK}\left(\theta \right)$$

where *ε* is the parity of the reactive system. *K* and *M* are the projection of *J* on the *z*-axis of the body-fixed and space-fixed representations, respectively. $D_{MK}^{J\varepsilon }\left(\Omega \right)$ is the normalized Wigner rotation matrix, which only depends on the Euler angle Ω. In this work, an improved L-shaped grid scheme [54], recently developed by Buren et al., is applied to improve the efficiency of numerical calculations, which decompose the total wavefunction into the interaction region the asymptotic region, and different numbers of rovibrational basis sets are used in the two regions. In the SF representation, the initial wave packet with a defined initial state (*v*_0_, *j*_0_, *l*_0_) can be prepared in the reactant asymptote:

(9) $$\Psi_{v_{0}j_{0}l_{0}}^{JMp}(t=0)=G(R)\phi_{v_{0}j_{0}}(r)|JMj_{0}l_{0}\varepsilon \rangle$$

where *G*(*R*) is a Gaussian function, $\phi_{v_{0}j_{0}}\left(r\right)$ is the eigenfunction of the diatom Hamiltonian, and $|JMj_{0}l_{0}\varepsilon \rangle$ is the total angular momentum eigenfunction. The state-to-state scattering matrix *S^J^* is extracted by the reactant coordinate-based method [62,63], and the second-order split operator propagator [64] is selected to evaluate the wave packet. The state-to-state reaction probability can be calculated as:

(10) $$P_{v\prime j\prime \leftarrow v_{0}j_{0}}^{J}=\frac{1}{2j_{0}+1}{\sum_{K,K_{0}}\left|S_{\nu \prime j\prime K\leftarrow \nu_{0}j_{0}K_{0}}^{J}\right|}^{2}$$

The state-to-state ICSs are obtained by summing over all available partial waves *J*, written as:

(11) $$\sigma_{v\prime j\prime \leftarrow v_{0}j_{0}}=\frac{\pi }{(2j_{0}+1)k_{v_{0}j_{0}}^{2}}\sum_{K}\sum_{K_{0}}{\sum_{J}\left(2J+1\right)\left|S_{\nu \prime j\prime K\leftarrow \nu_{0}j_{0}K_{0}}^{J}\right|}^{2}$$

where *E_c_* presents the collision energy and $k_{v_{0}j_{0}}=\sqrt{2\mu_{R}E_{c}}$ is the wave vector in the entrance channel. The state-to-state DCSs are calculated by:

(12) $$\frac{d\sigma_{vj\leftarrow v_{0}j_{0}}(\vartheta ,E)}{d\Omega }=\frac{1}{\left(2j_{0}+1\right)}{\sum_{K}\sum_{K_{0}}\left|\frac{1}{2ik_{v_{0}j_{0}}}\sum_{J}\left(2J+1\right)d_{KK_{0}}^{J}(\vartheta )S_{vjK\leftarrow v_{0}j_{0}K_{0}}^{J}\right|}^{2}$$

where ϑ is the scattering angle and $d_{KK_{0}}^{J}(\vartheta )$ expresses the element of reduced Wigner rotation matrix.

In this work, the initial rovibrational state of the reactant H_2_ and D_2_ molecules is set at *v*_0_ = 0 and *j*_0_ = 0. A total of 80 and 99 partial waves are calculated for the Be^+^(^2^S) + H_2_(*v*_0_ = 0, *j*_0_ = 0) and Be^+^(^2^S) + D_2_(*v*_0_ = 0, *j*_0_ = 0) reactions, respectively, which can yield converged ICSs and DCSs for the collision energy below 4.0 eV. The main numerical parameters used in the TDWP calculations are listed in Table 2 by numerous convergence tests.

## 4. Conclusions

In this study, a globally accurate ground-state BeH_2_^+^ PES is constructed by combining the high-level ab initio calculations and the NN model. A total of 18,657 energy points, calculated by the icMRCI + Q method with aug-cc-PVQZ basis set, are selected to participate in the NN fitting and the fitting RMSE is only 1.03 meV. The molecular constants of H_2_(X^1^Σ_g_^+^) and BeH^+^(X^1^Σ_g_) molecules calculated on the NN PES are in good agreement with the experimental data, and the PES can accurately reproduce the structure and energy of equilibrium configuration. The topography characteristics of the PES are described in detail, and there exist multiple wells and barriers on the PES. Based on the newly constructed PES, the state-to-state quantum dynamics of the Be^+^(^2^S) + H_2_(*v*_0_ = 0, *j*_0_ = 0) → BeH^+^ + H and Be^+^(^2^S) + D_2_(*v*_0_ = 0, *j*_0_ = 0) → BeD^+^ + D reactions are carried out using the TDWP method to study the microscopic dynamics processes and isotope substitution effects. The reaction probability curves for the low partial waves present some oscillation structures due to the forming of a triatomic complex in the well on the global MEP, and the resonances are smoothed at high partial waves because of the effects of centrifugal potential. The total ICS values of the Be^+^(^2^S) + D_2_ reaction are smaller than that of the Be^+^(^2^S) + H_2_ reaction when the collision energy is below 4.0 eV owing to the larger endothermicity of the former. The product molecules of the two reactions prefer to distribute at low vibrational and high rotational states at relatively low collision energy, and there is obvious vibrational population inversion as the increase of collision energy, especially for the BeD^+^ product channel. The total DCSs of the two reactions present the forward-backward symmetry angle distributions at relatively low collision energy, whereas the backward scattering gradually plays the dominant role as the collision energy increases. The calculated dynamics results suggest that the two reactions follow the complex-forming mechanism when the collision energy is slightly larger than the corresponding threshold, whereas the direct abstraction process gradually plays the dominant role at relatively high collision energy. In addition, the isotope substitution has little influence on the dynamics features of the Be^+^(^2^S) + H_2_, except for the difference in the dynamics values.

## Figures and Tables

**Table 1:** Molecular constants of H_2_(X^1^Σ_g_^+^) and BeH^+^(X^1^Σ^+^) molecules.

		NN PES	Experimental Results
H_2_(X^1^Σ_g_^+^)	*r_e_* (*a*_0_)	1.402	1.401
	*D_e_* (eV)	4.741	4.747
	*ω_e_* (cm^−1^)	4391.2	4401.2
	*ω_e_x_e_* (cm^−1^)	121.93	121.33
BeH^+^(X^1^Σ^+^)	*R_e_* (Bohr)	2.487	2.480
	*D_e_* (eV)	3.157	3.280
	*ω_e_* (cm^−1^)	2222.7	2221.7
	*ω_e_x_e_* (cm^−1^)	39.75	39.79

**Table 2:** Numerical parameters used in the TDWP calculations.

	**Be^+^**(^2^S) **+ H_2_**	**Be^+^**(^2^S) **+ D_2_**
Grid ranges and sizes	*R* ∈ [0.1, 30.0], $N_{R}^{\mathrm{tot}}$ = 349, $N_{R}^{int}$ = 139	*R* ∈ [0.1, 30.0], $N_{R}^{\mathrm{tot}}$ = 499, $N_{R}^{int}$ = 199
	*r* ∈ [0.4, 25.0], $v_{int}$ = 249, $v_{asy}$ = 9	*r* ∈ [0.4, 25.0], $v_{int}$ = 249, $v_{asy}$ = 11
	$j_{int}$ = 149, $j_{asy}$ = 29	$j_{int}$ = 139, $j_{asy}$ = 29
Initial wave packet	*R*_c_ = 15.0 Δ_R_ = 0.28. *k*_0_ = (2*E*_0_*μ_R_*)^1/2^ with *E*_0_ = 2.6 eV	*R*_c_ = 15.0 Δ_R_ = 0.28 *E*_0_ = 2.6 eV
Propagation time	15,000, Δ*_t_* = 10	15,000, Δ*_t_* = 10
Highest *J* value	80	99

## Data Availability

The data presented in this study are available on request.
